# Baroreflex sensitivity predicts therapeutic effects of metoprolol on pediatric postural orthostatic tachycardia syndrome

**DOI:** 10.3389/fcvm.2022.930994

**Published:** 2022-09-14

**Authors:** Yaxi Cui, Yuanyuan Wang, Ping Liu, Yuli Wang, Junbao Du, Hongfang Jin

**Affiliations:** ^1^Department of Pediatrics, Peking University First Hospital, Beijing, China; ^2^Key Laboratory of Molecular Cardiovascular Sciences, Ministry of Education, Beijing, China

**Keywords:** children, postural orthostatic tachycardia syndrome, baroreflex sensitivity, metoprolol, effectiveness, predictor

## Abstract

**Objective:**

To determine if the baseline baroreflex sensitivity (BRS) could be a useful predictor for the metoprolol therapeutic efficacy on postural orthostatic tachycardia syndrome (POTS) in children.

**Methods:**

In this retrospective case-control study, 54 children suffering from POTS treated with metoprolol were recruited from the pediatric department of Peking University First Hospital. After 2–3 months of metoprolol treatment, all subjects were divided into responders and non-responders based on whether the symptom score (SS) was decreased by over 50% after metoprolol treatment at the follow-up. The baseline demographic parameters and the supine BRS during the head-up tilt test (HUTT) obtained by Finapres Medical System (FMS) were compared between the two groups. The value of BRS to predict the effectiveness of POTS was analyzed by a receiver-operating characteristic (ROC) curve.

**Results:**

The age, sex, height, weight, body mass index (BMI), course of the disease, baseline SS, medication time, metoprolol dose, and follow-up time of the subjects were not statistically different between the responders and non-responders (*P* > 0.05). The decline in symptom scores (ΔSS) of the responders was more obvious than that of the non-responders (*P* < 0.01). The supine BRS, BRS at maximum HR, supine heart rate (HR), and maximum HR were different between responders and non-responders (*P* < 0.01, *P* = 0.022, *P* < 0.01, *P* = 0.047). The binary multivariable analysis showed that baseline supine BRS was significantly associated with the response to metoprolol therapy [OR: 2.079, 95% CI: (1.077, 4.015), *P* = 0.029]. According to the ROC curve, the area under the curve (AUC) of baseline BRS was 0.912 (95% CI, 0.840–0.984), with a cut-off value of 8.045 ms/mmHg, yielding a sensitivity and specificity of 75.8% and 95.2%, respectively, in predicting the effectiveness of POTS.

**Conclusion:**

The baseline supine BRS level > 8.045 ms/mmHg can predict a good therapeutic response to metoprolol and the results would assist in guiding the individualized β-adrenoceptor blocker use in pediatric patients suffering from POTS.

## Introduction

Postural orthostatic tachycardia syndrome (POTS) is a subtype of autonomic disorder in children and described as an abnormally fast heart rate (HR) with chronic orthostatic intolerance (OI) symptoms associated with postural change (1). Its symptoms include dizziness, weakness, tachycardia, headache, fatigue, nausea, abdominal pain, and even syncope (2, 3). POTS appears to become more common in children and adolescents (4). According to a study in 2014, the prevalence of POTS was 6.8%, and the data suggested that some severe cases of POTS experienced syncope in children (5, 6). Due to its debilitating nature, 40–50% of children with POTS could not go to school on a regular basis. The quality of life and the quality of learning of pediatric patients are negatively affected (7). Therefore, early appropriate management of POTS can assist children in improving their quality of life.

Up to now, the mechanisms underlying POTS have been incompletely understood, but the pathogenic factors such as increased sympathetic tone, hypovolemia, and vasomotor dysfunction might contribute to the symptoms of POTS (8–10). POTS management recommendations in the guidelines involve non-pharmacologic and pharmacologic approaches, such as increased sodium and water intake, physical exercise training, β-adrenoceptor blocker, pyridostigmine, fludrocortisone, and midodrine hydrochloride (4, 11). Theoretically, the clinical treatment should depend on the mechanisms for POTS. Metoprolol, one of the most prescribed β-adrenoceptor blockers, targets the adrenergic receptor to lessen the symptoms of pediatric patients with POTS with increased sympathetic activity as the main pathogenic mechanism. Therefore, due to the complex and diverse mechanisms of POTS including excessive sympathetic tone, hypovolemia, and vasomotor dysfunction (10), β-adrenoceptor blockers would be effective for those suffering from POTS with an excessive sympathetic tone other than those with other pathogenic mechanisms. Therefore, looking for useful predictors reflecting the excessive sympathetic tone that patients with POTS might have as the main pathogenic mechanism would be a critical and challenging clinical issue necessary to assist in selecting the use of the β-adrenoceptor blocker in children with POTS before treatment.

Baroreflex regulation is an important reflex adjustment in the human body that enables the circulatory system to adapt to varying conditions in daily life, while maintaining stable blood pressure, HR, and blood volume within a physiologic range (12). The effectiveness of the baroreflex can be reflected by baroreflex sensitivity (BRS). BRS represents a function of cardiovascular baroreceptor reflex, and abnormal changes in baroreflex have been identified as a key mechanism for POTS (6, 9). When standing up from the supine position, approximately 500 ml of the blood volume is redistributed to the lower limbs due to gravity in adults (13). A similar redistribution occurs in children, but the volume of blood redistributed is unknown. Therefore, the venous return and stroke cardiac output are reduced instantly, resulting in a reduced frequency of the transmission of the impulses *via* carotid sinus and aorta arch baroreceptors to nucleus tractus solitarius, leading to unloading of vagal tone and increased sympathetic efferent impulses. The increased peripheral resistance and HR *via* the center of cardiovascular regulation could counter the initially decreased blood pressure (14). Most authors consider BRS as a measure of sympathetic modulation (15, 16). Therefore, if a patient suffering from POTS has an increased BRS, representing an over-enhanced sympathetic tone as a possible mechanism, metoprolol treatment should be used. As such, we presumed that POTS children with increased BRS levels before treatment might have a good response to metoprolol.

Therefore, this study aimed to explore whether BRS could predict the therapeutic effects of metoprolol on pediatric patients with POTS to find out a useful indicator to assist in implementing β-adrenoceptor blocker-based individualized therapy for pediatric patients with POTS.

## Materials and methods

### Study participants

A total of 54 children aged 12.6 ± 2.7 years with POTS and treated with metoprolol between November 2013 and June 2021 were recruited. All parents of the study participants provided consent statements. The Peking University First Hospital Ethics Committee in Beijing, China, gave its approval to the study.

The following criteria were used to diagnose POTS in children: (1) patients had chronic OI, such as chest tightness, dizziness, fatigue, and palpitation; (2) the chronic symptoms lasted for at least 3 months; (3) HR was increased by ≥ 40 bpm or maximum HR ≥ 130 bpm (in children 6–12 years old) or ≥ 125 bpm (in adolescents 13–18 years old) when patients changed position to upright position from supine within 10 min; and (4) other diseases, namely, metabolic diseases, cardio-cerebrovascular diseases, and psychogenic disorders were excluded (6, 17–19).

### Head-up tilt test

The Head-up tilt test (HUTT) was performed based on the previously published literature (17, 18). All study subjects were required to empty their bladder after fasting overnight. The test environment was quiet, warm, and dim light. The study subjects were required to lay on a tilt table (SHUT-100A, Standard, and Hut-821, Juchi, China) for at least 10 min, and HR, blood pressure, and electrocardiograph of children were continuously monitored. Then, the tilt table was tilted to 60 degrees, and the HR, blood pressure, and electrocardiograph of children were continuously monitored. The test was terminated if the positive response appeared, or otherwise 45-min duration was completed if the positive response did not appear.

### The clinical data collection and symptom score calculation

All patients’ clinical demographic data were recorded, including age, height, weight, and body mass index (BMI). The following clinical symptoms were recorded for the symptom score (SS) calculation: syncope, nausea, dizziness, chest tightness, headache, palpitation, blurred vision, cold sweat, hand tremors, and inattention. The SS was calculated based on the symptom frequency, which was used to assess the effectiveness of the metoprolol. The following specific scoring calculation criteria were as follows (18, 20): 0 point indicated no symptoms occurred; 1 point, symptoms occurred at most once per month; 2 points, symptoms occurred 2–4 times per month; 3 points, symptoms occurred 2–7 times per week; and 4 points, symptoms occurred at least once per day. The total of individual SS based on the 10 symptoms was used to calculate the final score. Those whose scores at the end of follow-up were decreased by over 50% compared to their baseline SS at the time of diagnosis were considered responders, while the others were considered non-responders (21).

### Baroreflex sensitivity measurement

All BRS measurements were obtained by Finapres Medical System-FMS (Finometer PRO, FMS Company, Netherlands). The BP and beat-by-beat R-R interval were continuously monitored through the finger sensor of the FMS. Arterial BRS was measured continuously and non-invasively by a time-domain sequential method with an inflated finger cuff. The device was connected to the finger cuff to detect the changes in blood pressure and cardiac output. Through the mathematical model in the software, the arm blood pressure pulse waveform was generated from the finger blood pressure waveform. The BRS at resting and tilt positions was calculated through this method (6, 22).

### Treatment and follow-up

**FIGURE 1:**
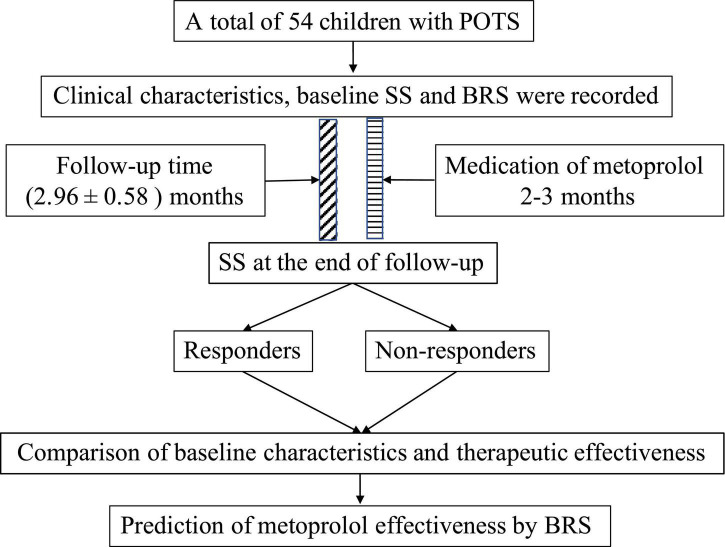
Flowchart of the study. POTS, postural orthostatic tachycardia syndrome; SS, symptom score; BRS, baroreflex sensitivity.

All participants in this study after the diagnosis were given metoprolol at 0.5 mg/(kg ⋅ d) in two divided doses, and not > 2 mg/(kg ⋅ d). All patients took their medication for about 2–3 months. No patients were dropped out of the study due to the poor effect of metoprolol or the occurrence of side effects. All participants completed the follow-up after their medications of metoprolol were completed and the mean follow-up time was 2.96 ± 0.58 months. The symptoms and adverse effects prior to the medication and at the follow-up were recorded by a professional doctor in the pediatric cardiology clinic or over the telephone (Figure 1). The baseline SS prior to the treatment and at the end of follow-up were calculated, and thus the participants were defined as the responders and non-responders according to the criteria mentioned above. For the non-responders, we replaced the treatment with empirical medications, namely, oral rehydration salt (ORS), midodrine hydrochloride, and metoprolol plus midodrine hydrochloride.

### Statistical analysis

The data were analyzed by SPSS statistics, version 25.0 (IBM, NY, USA). The normal continuous variables were expressed as mean ± standard deviation or median (25th and 75th percentiles) according to the Shapiro–Wilk test. The intergroup comparison was analyzed by independent *t*-test or Mann–Whitney *U*-test. The categorical variables were expressed as the number of cases and analyzed by the Chi-square test. A binary logistic regression model with a 95% confidence interval was used to analyze the factors affecting the therapeutic effect. Candidate variables with a significant difference between the responder and non-responder groups (*P*-value < 0.05) on univariate analysis were included in the multivariable model. The predictive value of BRS in patients with POTS was assessed by the receiver-operating characteristic (ROC) curve. The optimal cut-off was calculated using the Youden index. The area under curve (AUC) of 0.5–0.7 indicated a low predictive value, 0.7–0.9 indicated a moderate predictive value, and > 0.9 indicated a high predictive value. *P* < 0.05 was considered statistically significant.

## Results

### The demographic parameters between the responders and non-responders

At the follow-up after metoprolol treatment, among the 54 patients, 33 patients were evaluated as responders, while 21 as non-responders. The demographic data including age, sex, height, weight, and BMI did not differ between the responders and non-responders (*P* > 0.05, Table 1).

**TABLE 1:** Comparison of baseline characteristics between responders and non-responders.

Variable	Responders	Non-responders	Statistic	*P*-value
Sex (M/F)	14/19	11/10	0.512	0.474
Age (year)	12.6 ± 2.3	12.5 ± 3.2	−0.110	0.913
Body height (cm)	159.1 ± 11.1	156.7 ± 17.5	0.768	0.446
Body weight (kg)	54.2 ± 20.9	53.6 ± 19.3	0.388	0.701
BMI (kg/m^2^)	20.4 ± 3.7	21.3 ± 5.5	0.676	0.504
Baseline symptom score	8 (5, 12)	6 (4, 10)	0.597	0.550
Symptom score after treatment	1 (0, 3)	4 (2.5, 7)	−3.595	<0.01
Δ Symptom score	6 (4, 9)	2 (1, 4.5)	4.215	<0.01
Duration of disease (month)	9 (3, 14.5)	12 (4, 24)	−0.978	0.328
Drug dose (mg/d)	22.15 ± 4.96	21.73 ± 8.75	0.206	0.838
Medication time (month)	2.76 ± 0.61	2.29 ± 0.96	2.013	0.053
Follow-up time (month)	3.06 ± 0.66	2.81 ± 0.40	1.568	0.123
Supine BRS (ms/mmHg)	15.98 (7.81, 20.11)	4.63 (3.39, 6.30)	5.066	<0.01
Supine HR (bpm)	75.21 ± 12.30	93.61 ± 20.29	−3.667	<0.01
Supine SBP (mmHg)	111.03 ± 14.02	108.07 ± 16.98	0.688	0.495
Supine DBP (mmHg)	64.33 ± 10.38	65.91 ± 14.01	−0.469	0.641
BRS at max HR (ms/mmHg)	3.99 (2.74, 6.19)	2.85 (2.27, 3.55)	2.298	0.022
Max HR (bpm)	115.36 ± 12.63	123.00 ± 14.20	0.389	0.047

BMI, body mass index; Δ symptom score, the decline of symptom score = baseline symptom score—symptom score after treatment; BRS, baroreflex sensitivity; HR, heart rate; SBP, systolic blood pressure; DBP, diastolic blood pressure; Max HR, maximum heart rate during Head-up tilt test.

### The symptom scores between responders and non-responders

There were no significant differences in the baseline SS, and the duration of disease, drug dose, medication time, and follow-up time between responders and non-responders. The SS after the treatment of responders was lower than that of the non-responders [1 (0, 3) vs. 4 (2.5, 7), *P* < 0.01]. The decline in symptom scores (ΔSS) of the responders was more obvious than that of the non-responders (*P* < 0.01) (Table 1).

### The hemodynamic parameters between the responders and non-responders

The supine HR before treatment in the responders was significantly lower than that in the non-responders (75.21 ± 12.30 bpm vs. 93.61 ± 20.29 bpm, *P* < 0.01). There were no statistical differences in systolic blood pressure (SBP) and diastolic blood pressure (DBP) before treatment between the two groups (*P* > 0.05). A significant higher baseline BRS was found in the responders than in the non-responders [15.98 (7.81, 20.11) ms/mmHg vs. 4.63 (3.39, 6.30) ms/mmHg, *P* < 0.01]. The BRS at maximum HR during HUTT in the responders was significantly higher than that in the non-responders [3.99 (2.74, 6.19) ms/mmHg vs. 2.85 (2.27, 3.55) ms/mmHg, *P* = 0.022]. The maximum HR during HUTT in the responders was significantly lower than that in the non-responders (115.36 ± 12.63 bpm vs. 123.00 ± 14.20 bpm, *P* = 0.047) (Table 1).

### Factors affecting therapeutic efficacy of metoprolol by multivariable analysis

Baseline supine BRS, supine HR, BRS at maximum HR during HUTT, and maximum HR during HUTT with a *P*-value < 0.05 on univariate analysis were included in the binary multivariable model to analyze the therapeutic efficacy of metoprolol. The results showed that the baseline supine BRS was significantly associated with the response to metoprolol therapy [OR: 2.079, 95% CI: (1.077, 4.015), *P* = 0.029, Table 2]. The association also significantly remained after adjusting for age, sex, and BMI [OR: 2.017, 95% CI: (1.228, 3.315), *P* = 0.006].

**TABLE 2:** Multivariable logistic regression analysis of therapeutic response to metoprolol.

Variables	B	SE	Wald	*P*-value	Exp (B)	95% CI
Baseline BRS (ms/mmHg)	0.732	0.336	4.758	0.029	2.079	(1.077, 4.015)
HR (bpm)	−0.032	0.033	0.996	0.318	0.968	(0.908, 1.032)
BRS at max HR	−0.161	−0.390	0.171	0.679	0.851	(0.397, 1.827)
Max HR	−0.003	0.039	0.007	0.934	0.997	(0.923, 1.076)

BRS, Baroreflex sensitivity; HR, heart rate; Max HR, maximum heart rate during HUTT; Exp (B), the exponentiation of the B coefficient; 95% CI, 95% confidence interval.

### The predictive value of supine baroreflex sensitivity for metoprolol therapeutic efficacy

**FIGURE 2:**
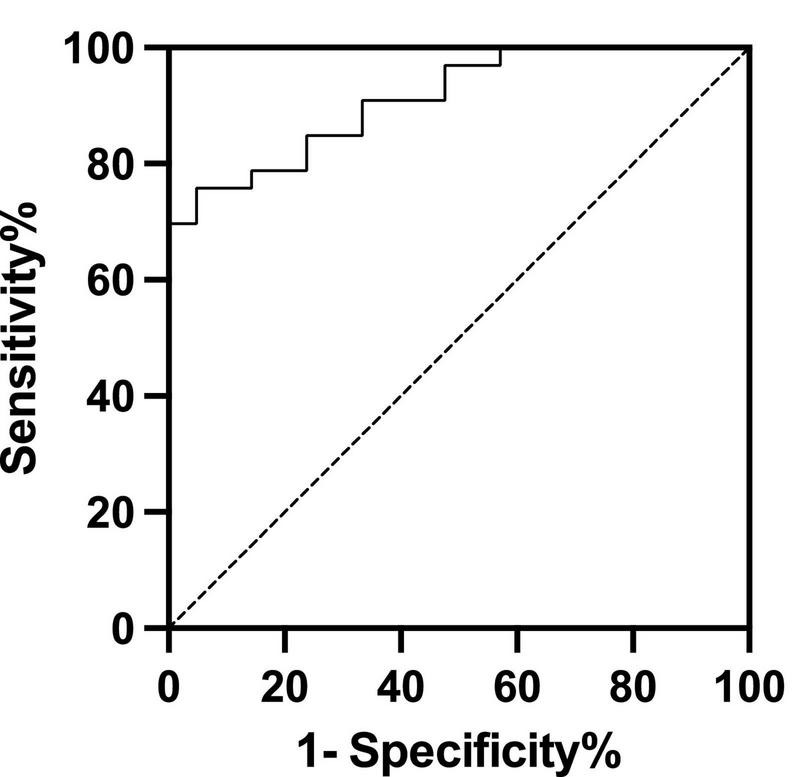
Receiver operating characteristic curve of baseline baroreflex sensitivity levels for predicting the therapeutic response to metoprolol. The area under the curve was 0.912 (95% CI: 0.840–0.984; *P* < 0.01). Using a cut-off value for baseline baroreflex sensitivity of 8.045 ms/mmHg yielded both sensitivity of 75.8% and specificity of 95.2% in predicting the efficacy of metoprolol on postural orthostatic tachycardia syndrome. The *y*-axis represents the sensitivity to predict the effectiveness of different BRS levels in metoprolol therapy. The *x*-axis represents the false-positive rate (1-specificity) of the prediction.

The cut-off value of supine BRS to predict the efficacy of metoprolol on pediatric POTS was 8.045 ms/mmHg, yielding a sensitivity and a specificity of 75.8% and 95.2%, respectively, with an AUC of 0.912 (95% CI: 0.840–0.984; *P* < 0.01, Figure 2).

## Discussion

In this study, we, for the first time, showed that the responders to metoprolol treatment had higher supine BRS than non-responders with POTS in children. The baseline supine BRS was associated with the therapeutic efficacy of metoprolol. The ROC curve indicated that the baseline supine BRS could be a useful predictor of the therapeutic response to metoprolol in pediatric patients suffering from POTS. The supine BRS cut-off > 8.045 ms/mmHg successfully predicted the effective therapeutic response to metoprolol treatment with predictive sensitivity and specificity of 75.8% and 95.2%, respectively.

The clinical OI symptoms caused by POTS affect the daily life of the children. Also, it may cause substantial disability in children when syncope occurs (23). Failure to recognize the heterogeneous mechanisms for POTS contributes to the difficulties in the management of patients (24). The drug treatment should be individualized, based on the mechanisms for the disorder. POTS is characterized by an abnormal increase of HR as the body position changes from supine to upright and sometimes is associated the OI symptoms (25, 26). When changing the position from supine to upright, blood volume was instantaneously shifted to the abdomen and lower extremities to a certain extent due to gravity (9). The redistribution of blood within the circulatory system forced by gravity decreases arterial blood pressure, resulting in reduced afferent impulses of arterial baroreceptors. Arterial baroreceptors in the carotid sinuses and the region of the aortic arch can sense the downward changes in BP and lead to a decreased parasympathetic tone and an increased sympathetic activity, causing increased HR, myocardial contraction, cardiac output, peripheral resistance, and vascular constriction (15). Although the underlying mechanisms for POTS are heterogeneous, excessively increased sympathetic activity is one of the most contributing factors to POTS. The metoprolol works mainly by slowing down the HR and improving some OI symptoms by blocking the myocardial β-adrenoceptor to inhibit the sympathetic nervous activity (27, 28).

How to find out the biomarkers to identify the children with POTS whose main pathogenesis is over-activated sympathetic nerve tone and thus treat them with metoprolol is the major clinical issues that need to be solved. Several biomarkers were indicated to be useful in reflecting the over-activated sympathetic activity to help in selecting metoprolol for pediatric patients with POTS. Zhao et al. discovered that using the plasma copeptin level can predict the effects of metoprolol in pediatric patients suffering from POTS (29). Zhang et al. used baseline orthostatic plasma norepinephrine levels to predict the efficacy of metoprolol on POTS as plasma norepinephrine reflects the hyperadrenergic state in patients (30). Lin et al. compared the difference of plasma C-type natriuretic peptide (CNP) between metoprolol responders and non-responders and found that plasma CNP could be a reliable indicator as it could stimulate the synthesis of catecholamine (31, 32). However, the detection of these biomarkers requires blood samples, and the invasive venous blood sample collection is often difficult for children to accept. Also, the orthostatic plasma norepinephrine level is very unstable, thus limiting the clinical use of this indicator. Wang et al. discovered that a combination of baseline triangular (TR) with the standard deviation of all normal-to-normal intervals (SDNN) index might be a measure to assess the metoprolol efficacy (18). But, the TR and SDNN index need 24 h dynamic Holter electrocardiogram, and the results are not immediately available. Therefore, its clinical application is limited to some extent. The BRS had higher sensitivity and specificity to predict the efficacy of metoprolol on POTS in children and adolescence than HR and HR differences during HUTT which was reported by Wang et al. (27).

This study first showed that the baseline supine BRS might be a useful predictor for effective metoprolol treatment in pediatric patients with POTS. The baseline supine BRS has unique advantages, such as its non-invasiveness, easy operation, and high specificity. The difference in BRS indicated the role of baroreceptors reflex in POTS and help us in revealing the pathogenic mechanism underlying POTS. Therefore, the baseline supine BRS might be a useful predictor of the therapeutic efficacy of metoprolol on POTS.

The SS used to evaluate the symptoms of patients with POTS in our study was validated in previous studies and proved to be a useful and practical method (21). Chen et al. (21) revealed that OI patient with a reduced SS over 50% after treatment was regarded as a responder to the certain treatment.

However, this study is a single-center retrospective study and the case number is not large enough. In the future, large sample-sized and multicenter-based prospective studies will be needed to further validate the predicting value of baseline supine BRS in the selection of metoprolol treatment for pediatric POTS, assisting in implementing the individualized therapy of metoprolol for pediatric POTS.

Collectively, our study showed that the responders to metoprolol treatment had higher baseline supine BRS than non-responders with POTS in children. The supine BRS was associated with the therapeutic efficacy of metoprolol. The baseline supine BRS level > 8.045 ms/mmHg predicted a good therapeutic response to metoprolol and the results would assist in guiding the individualized β-adrenoceptor blocker use in pediatric patients suffering from POTS.

## Data availability statement

The raw data supporting the conclusions of this article will be made available by the authors, without undue reservation.

## Ethics statement

The studies involving human participants were reviewed and approved by the Peking University First Hospital Ethics Committee in Beijing, China. Written informed consent to participate in this study was provided by the participants’ legal guardian/next of kin.

## Author contributions

YC collected and analyzed the data, drafted the initial manuscript, interpreted the result, and critically revised the manuscript. YYW collected the data and revised the manuscript. PL and YLW performed the head-up tilt test and reviewed the manuscript. JD and HJ conceptualized and designed the study and critically reviewed and revised the manuscript. All authors agreed to accept responsibility for this work and agreed on the final manuscript as submitted.

## References

[1] Brignole M Moya A de Lange FJ Deharo JC Elliott PM Fanciulli A 2018 ESC Guidelines for the diagnosis and management of syncope. *Eur Heart J.* (2018) 39:1883–948. 10.1093/eurheartj/ehy037 29562304

[2] Boris JR Bernadzikowski T. Demographics of a large paediatric postural orthostatic tachycardia syndrome program. *Cardiol Young.* (2018) 28:668–74. 10.1017/s1047951117002888 29357955

[3] Boris JR. Postural orthostatic tachycardia syndrome in children and adolescents. *Auton Neurosci.* (2018) 215:97–101. 10.1016/j.autneu.2018.05.004 29778304

[4] Raj SR Guzman JC Harvey P Richer L Schondorf R Seifer C Canadian cardiovascular society position statement on postural orthostatic tachycardia syndrome (POTS) and related disorders of chronic orthostatic intolerance. *Can J Cardiol.* (2020) 36:357–72. 10.1016/j.cjca.2019.12.024 32145864

[5] Lin J Han Z Li X Ochs T Zhao J Zhang X Risk factors for postural tachycardia syndrome in children and adolescents. *PLoS One.* (2014) 9:e113625. 10.1371/journal.pone.0113625 25474569 PMC4256207

[6] Li H Liao Y Wang Y Liu P Sun C Chen Y Baroreflex sensitivity predicts short-term outcome of postural tachycardia syndrome in children. *PLoS One.* (2016) 11:e0167525. 10.1371/journal.pone.0167525 27936059 PMC5147897

[7] Moon J Kim DY Byun JI Sunwoo JS Lim JA Kim TJ Orthostatic intolerance symptoms are associated with depression and diminished quality of life in patients with postural tachycardia syndrome. *Health Qual LIfe Outcomes.* (2016) 14:144. 10.1186/s12955-016-0548-x 27729043 PMC5059908

[8] Garland EM Celedonio JE Raj SR. Postural tachycardia syndrome: beyond orthostatic intolerance. *Curr Neurol Neurosci Rep.* (2015) 15:60. 10.1007/s11910-015-0583-8 26198889 PMC4664448

[9] Raj SR. Postural tachycardia syndrome (POTS). *Circulation.* (2013) 127:2336–42. 10.1161/CIRCULATIONAHA.112.144501 23753844 PMC3756553

[10] Raj SR Black BK Biaggioni I Paranjape SY Ramirez M Dupont WD Propranolol decreases tachycardia and improves symptoms in the postural tachycardia syndrome: less is more. *Circulation.* (2009) 120:725–34. 10.1161/circulationaha.108.846501 19687359 PMC2758650

[11] Cutsforth-Gregory JK Sandroni P. Clinical neurophysiology of postural tachycardia syndrome. *Handb Clin Neurol.* (2019) 161:429–45. 10.1016/b978-0-444-64142-7.00066-7 31307619

[12] Hainsworth R. Cardiovascular control from cardiac and pulmonary vascular receptors. *Exp Physiol.* (2014) 99:312–9. 10.1113/expphysiol.2013.072637 24058186

[13] Raj SR. The postural tachycardia syndrome (POTS): pathophysiology, diagnosis & management. *Indian Pacing Electrophysiol J.* (2006) 6:84–99. 10.1111/joim.12852 16943900 PMC1501099

[14] Kaufmann H Norcliffe-Kaufmann L Palma JA. Baroreflex dysfunction. *N Engl J Med.* (2020) 382:163–78. 10.1056/NEJMra1509723 31914243

[15] La Rovere MT Pinna GD Raczak G. Baroreflex sensitivity: measurement and clinical implications. *Ann Noninvasive Electrocardiol.* (2008) 13:191–207. 10.1111/j.1542-474X.2008.00219.x 18426445 PMC6931942

[16] Hissen SL Sayed KE Macefield VG Brown R Taylor CE. The stability and repeatability of spontaneous sympathetic baroreflex sensitivity in healthy young individuals. *Front Neurosci.* (2018) 12:403. 10.3389/fnins.2018.00403 29962929 PMC6010576

[17] Wang C Li Y Liao Y Tian H Huang M Dong X 2018 Chinese pediatric cardiology society (CPCS) guideline for diagnosis and treatment of syncope in children and adolescents. *Sci Bull.* (2018) 63:1558–64. 10.1016/j.scib.2018.09.019 36751076

[18] Wang Y Zhang C Chen S Liu P Wang Y Tang C Heart rate variability predicts therapeutic response to metoprolol in children with postural tachycardia syndrome. *Front Neurosci.* (2019) 13:1214. 10.3389/fnins.2019.01214 31780890 PMC6861190

[19] Zhao J Han Z Zhang X Du S Liu AD Holmberg L A cross-sectional study on upright heart rate and BP changing characteristics: basic data for establishing diagnosis of postural orthostatic tachycardia syndrome and orthostatic hypertension. *BMJ Open.* (2015) 5:e007356. 10.1136/bmjopen-2014-007356 26033944 PMC4458681

[20] Yuan P Li X Tao C Du X Zhang C Du J Poincaré plot can be a useful tool to select potential responders to metoprolol therapy in children with vasovagal syncope. *Int J Gen Med.* (2022) 15:2681. 10.2147/IJGM.S352928 35300141 PMC8922042

[21] Chen L Wang L Sun J Qin J Tang C Jin H Midodrine hydrochloride is effective in the treatment of children with postural orthostatic tachycardia syndrome. *Circ J.* (2011) 75:927–31. 10.1253/circj.cj-10-0514 21301135

[22] Li H Liao Y Han Z Wang Y Liu P Zhang C Head-up tilt test provokes dynamic alterations in total peripheral resistance and cardiac output in children with vasovagal syncope. *Acta Paediatr.* (2018) 107:1786–91. 10.1111/apa.14342 29603793

[23] Vernino S Bourne KM Stiles LE Grubb BP Fedorowski A Stewart JM Postural orthostatic tachycardia syndrome (POTS): state of the science and clinical care from a 2019 National Institutes of Health Expert Consensus Meeting - Part 1. *Auton Neurosci.* (2021) 235:102828. 10.1016/j.autneu.2021.102828 34144933 PMC8455420

[24] Benarroch EE. Postural tachycardia syndrome: a heterogeneous and multifactorial disorder. *Mayo Clinic proc.* (2012) 87:1214–25. 10.1016/j.mayocp.2012.08.013 23122672 PMC3547546

[25] Jones PK Shaw BH Raj SR. Clinical challenges in the diagnosis and management of postural tachycardia syndrome. *Pract Neurol.* (2016) 16:431–8. 10.1136/practneurol-2016-001405 27660311

[26] Bryarly M Phillips LT Fu Q Vernino S Levine BD. Postural orthostatic tachycardia syndrome: JACC Focus Seminar. *J Am Coll Cardiol.* (2019) 73:1207–28. 10.1016/j.jacc.2018.11.059 30871704

[27] Wang S Zou R Cai H Wang Y Ding Y Tan C Heart rate and heart rate difference predicted the efficacy of metoprolol on postural tachycardia syndrome in children and adolescents. *J Pediatr.* (2020) 224:110–4. 10.1016/j.jpeds.2020.05.017 32464225

[28] Zhang FW Liao Y Li XY Chen L Jin HF Du JB. Therapies for postural tachycardia syndrome in children. *Zhonghua Er Ke Za Zhi.* (2011) 49:428–32. 10.3760/cma.j.issn.0578-1310.2011.06.008 21924055

[29] Zhao J Du S Yang J Lin J Tang C Du J Usefulness of plasma copeptin as a biomarker to predict the therapeutic effectiveness of metoprolol for postural tachycardia syndrome in children. *Am J Cardiol.* (2014) 114:601–5. 10.1016/j.amjcard.2014.05.039 24996552

[30] Zhang Q Chen X Li J Du J. Orthostatic plasma norepinephrine level as a predictor for therapeutic response to metoprolol in children with postural tachycardia syndrome. *J Transl Med.* (2014) 12:249. 10.1186/s12967-014-0249-3 25204388 PMC4177336

[31] Takekoshi K Ishii K Isobe K Nomura F Nammoku T Nakai T. Effects of natriuretic peptides (ANP, BNP, CNP) on catecholamine synthesis and TH mRNA levels in PC12 cells. *Life Sci.* (2000) 66:l303–11. 10.1016/s0024-3205(00)00549-x 10834306

[32] Lin J Han Z Li H Chen SY Li X Liu P Plasma C-type natriuretic peptide as a predictor for therapeutic response to metoprolol in children with postural tachycardia syndrome. *PLoS One.* (2015) 10:e0121913. 10.1371/journal.pone.0121913 25811760 PMC4374798

